# The Causal Effect of Gun Violence on Everyday Mobility Patterns Across US Neighborhoods

**DOI:** 10.1007/s40980-025-00139-1

**Published:** 2025-07-16

**Authors:** Karl Vachuska, Masoud Movahed

**Affiliations:** 1 University of Wisconsin–Madison, Madison, United States; 2 University of Pennsylvania, Philadelphia, United States; 3 Present Address: University of California, Santa Barbara, United States

**Keywords:** Mobility patterns, Racial inequality, Gun violence, Causal inference, Big data

## Abstract

Gun violence takes an enormous toll on neighborhoods and their residents in important ways. While research has identified that violence makes neighborhoods less appealing and livable, few studies have fully quantified the effect of violence on neighborhoods’ vitality and dynamism. In this study, we introduce the notion of ‘neighborhood activity,’ which we measure by the unique number of everyday visitors those neighborhoods receive from residents of *other* neighborhoods. Drawing on a large geographically-coded dataset of 30,000 gun violence incidents across US neighborhoods in conjunction with daily mobility pattern data based on 45 million mobile devices, we apply a quasi-experimental method to estimate the impact of gun violence on the number of visitors neighborhoods receive. We find that gun violence reduces neighborhoods’ visibility significantly, but its consequences are disproportionately distributed among non-White neighborhoods that are far less popular to begin with. Our estimation results indicate that gun violence cost neighborhoods approximately 9 million visitors in the year 2019 alone.

**Supplementary Information:**

The online version contains supplementary material available at 10.1007/s40980-025-00139-1.

## Introduction

The number of non-resident visitors to a neighborhood is often a strong indication of its vitality and dynamism. Visitors often contribute to capital circulation, economic dynamism, and expansion of social ties and networks, all of which are likely to yield positive material outcomes for the residents of a given neighborhood. An important body of scholarship underscores that neighborhood isolation limits access to employment, political influence, role models, and other material and non-material resources that are central to human capital development (Wilson, 1987). Isolation itself is directly linked to increased crime and violence, which then negatively impacts how individuals perceive neighborhoods (Cobbina et al., 2008). Violent crime is thus associated with higher residential turnover as well as lower real estate values—both of which are strong indicators of the *lack* of economic dynamism and vitality at the neighborhood level. At the micro-level, violent crime elicits avoidance strategies by individuals out of concern for safety and fear of victimization (Rader et al., 2007), including avoiding passing neighborhoods with higher levels of violent crime or walking at night, which then reinforces neighborhood isolation. These avoidance strategies are strongly tied to individuals’ perception of the risk of criminal behavior in a given neighborhood (Rader et al., 2007). Suffice it to say, violent crime has an acute, negative effect on the livability and desirability of neighborhoods across US cities (Cullen & Levitt, 1999; Hipp et al., 2009).

Previous research has shown that the extent to which individuals’ responses to criminal behavior in a given neighborhood vary is contingent upon a number of factors, including the gravity of the threat and individual characteristics such as race, gender, and age (Ferraro, 1995; Grohe et al., 2012; Kanan & Pruitt, 2002). While some researchers suggest that residents of particularly violent areas become desensitized to crime, others postulate that individuals residing in areas with more violent crime have more agile and neighborhood-specific discernments of where they should—or should not—go (Mrug et al., 2016). While some research suggests violence has no effect on mobility (Kerr et al., 2015), research generally suggests that violence has a negative effect on activity (Cuellar & Jung, 2025; Chiong et al., 2025).

Moreover, place-based racial composition often confounds perceptions of crime for those of the dominant racial group. In the US context, previous research has demonstrated that Whites in neighborhoods with higher proportions of Black residents tend to have significantly—often unreasonably so—high levels of fear of victimization (Pickett et al., 2012). Whites generally tend to have lower residential preferences for neighborhoods with higher proportions of non-Whites, specifically African Americans (Farley et al., 1994). This relationship is strongly mediated by Whites’ perception that neighborhoods with larger Black populations have greater crime rates and are unsafe (Harris, 2001; Krysan, 2002). But while previous research has indeed shown that White individuals are far less inclined to live in neighborhoods that have greater proportions of non-Whites, far less research has been conducted to conceptualize ‘neighborhood activity’ and its decline after witnessing a violent incident. Drawing on a novel dataset on mobility patterns, we bring forth a crucial dimension of neighborhood activity into the broader social stratification research. While research on residential preferences informs our expectations for non-resident preferences for visiting neighborhoods (Tokman, 2016), our approach to focus on unique visitors allows us to directly measure the effect of violent crime on neighborhood activity and vitality.

Residents’ visits to other neighborhoods across a city in their everyday life, including to both spatially proximal and non-proximal neighborhoods, is a crucial dimension of their *visibility*. The more a neighborhood is visible, the more likely it will see higher levels of economic dynamism and vitality. As Wheeler (2021) contends, economic activity within localities in the United States tends to be unevenly distributed, and business establishments pay attention to the characteristics of neighborhoods before settling in them (2006). One important way to gauge and measure neighborhood activity—and, to an extent, its popularity—is to identify the unique number of visitors it receives from other neighborhoods. Individuals’ social network, too, is likely to expand when they visit new neighborhoods. Levy et al., (2020, p. 928) argue that when individuals from other neighborhoods traverse a given area, they “forge qualitatively different forms of connectedness,” shaping local social dynamics. In contrast, neighborhood isolation can limit residents’ access to “social capital” (Wilson, 1987; Osterling, 2007; Levy et al., 2020). Osterling (2007, p. 125) theorizes that poor “socioeconomic characteristics” of neighborhoods can foster “inadequate social capital,” which, in turn, drives the broader phenomenon of disadvantaged neighborhood effects.

Altogether, there is strong evidence suggesting that individuals’ travel across neighborhoods in a city is *socially* patterned, which is to say, they disproportionately visit neighborhoods that are demographically similar to their residential neighborhoods (Krivo et al., 2013; Wang et al., 2018). To what extent the number of unique visitors by individuals from *other* neighborhoods—what we term and define as ‘neighborhood activity’—is affected by a gun violence incident in a given neighborhood? This is the research question that this paper takes up. Drawing on SafeGraph’s “Social Distance Metrics” dataset, which provides daily information on individuals’ visits, we estimate the effect of gun violence on neighborhood visibility measured by the number of unique visits individuals pay to a neighborhood after the violent incident.

### Violent Crime and Neighborhood Activity

Substantial research has detailed the effects of neighborhood disadvantage on a whole gamut of socioeconomic outcomes, including economic mobility (Chetty et al., 2018), educational attainment (Wodtke et al., 2016), and health outcomes (Sharkey & Faber, 2014), and cognitive ability (Sharkey & Elwert, 2011) among others (Sampson et al., 2002). Indeed, disparities in socioeconomic characteristics of neighborhoods correlate closely with numerous measures of neighborhood vitality and dynamism such as employment opportunities, collective efficacy, health and well-being outcomes (Sampson et al., 2002). Additionally, researchers consistently find that neighborhood disadvantage and racial segregation are associated with greater rates of homicide and violent crime (Massey & Denton, 1993; Peterson & Krivo, 2010; Sampson, 2012).

The findings of the previous research on neighborhood stratification are informative, but they also raise an important question: what is the effect of violent crime on neighborhood activity and vitality? Thanks to mobile devices, novel data on individuals’ mobility patterns has been made available, which we use to answer this question. Prior to examining the effect of violent crime on neighborhood vitality, it would be instructive to examine how the *lack* of neighborhood activity and dynamism is conceptually different than other forms of social isolation, including that which is based on race. Indeed, racial segregation, which signifies social isolation and systematic separation of people into racial or other ethnic groups across neighborhoods, is highly associated with higher crime rates (Massey & Denton, 1993; Peterson & Krivo, 2010; Sampson, 2012). However, lack of neighborhood activity is distinct from racial segregation; it is a measure of how *visible* and *dynamic* a neighborhood is for non-residents of that neighborhood in daily life. Hence, the lower number of visitors a given neighborhood receives, regardless of the underlying reasons, signifies less activity, visibility, and vitality and, therefore, more social isolation. Precisely for this reason, recent scholarship adds social isolation from affluent neighborhoods as yet another important dimension of neighborhood disadvantage (Levy et al., 2020). While “disadvantage” is often indexed by measures such as poverty, unemployment, and public assistance receipt, Levy and associates (2020) demonstrate that such resident-centered measures of disadvantage are inadequate and that we also need metrics of mobility-based disadvantage—what they term “Triple Disadvantage.” The frequency of unique visitors to a given neighborhood is, therefore, a strong indication of its dynamism and visibility. When residents from other neighborhoods visit a neighborhood throughout a city, especially those neighborhoods that are not spatially proximal, they forge a strong form of connectivity (Graif et al., 2017). This connectivity often yields positive effects for a whole gamut of socioeconomic outcomes, including local economic development, social capital, and networks, among others.

More importantly, neighborhood activity is also a racialized process. The racial gap that has already been identified across a wide range of socioeconomic outcomes such as employment, education, housing, and intergenerational economic mobility (Movahed & Neman, 2024; O’Brien et al., 2020; Quillian & Midtbøen, 2021), can also be found when looking at neighborhood visibility and activity. For example, there is strong evidence suggesting that individuals disproportionately visit neighborhoods that are demographically similar to their own residential neighborhoods (Krivo et al., 2013; Wang et al., 2018). Black and Hispanic residents are exposed to much higher rates of violent crime in their home neighborhoods relative to White individuals (Friedson & Sharkey, 2015; Peterson & Krivo, 2010). Moreover, racial residential segregation—and hence, social isolation—is a key driver of the Black-White homicide gap (Light & Thomas, 2019).

Relatedly, Jacobs’ (1961) concept of ’eyes on the street’ provides an important theoretical framework for understanding how neighborhood activity influences safety and crime rates. According to Jacobs, active public spaces, where residents and visitors engage with the environment, create informal social control that deters crime. When streets are populated, potential offenders are more likely to be observed, reducing the likelihood of criminal activity. Conversely, a decline in visitors—particularly in response to gun violence—can weaken this informal ‘supervision’ network, increasing the risk of further crime and social disorder. This aligns with broader research on urban decay and crime cycles, such as broken windows theory (Wilson & Kelling, 1982), which suggests that visible disorder and reduced social engagement contribute to escalating crime. Moreover, the racialized nature of mobility patterns plays a critical role in this dynamic process. Given that individuals tend to visit neighborhoods that align with their racial identity (Krivo et al., 2013; Wang et al., 2018), declines in visitor traffic due to violence may be especially pronounced in majority-Black and majority-Hispanic neighborhoods, further reinforcing their social and economic isolation. If neighborhoods experiencing gun violence become increasingly avoided, they may undergo a self-reinforcing cycle where diminished foot traffic leads to reduced informal social control, further entrenching racialized patterns of segregation and crime exposure. By incorporating Jacobs’ insights, this study situates neighborhood visibility and activity as not only markers of economic and social vitality but also as critical mechanisms shaping long-term crime trends and segregation.

While extensive research has examined the effects of neighborhood residential disadvantage on various outcomes, considerably less attention has been paid to a specific dimension of neighborhood effects: its activity and dynamism. We measure the notion of neighborhood activity by the frequency of *unique* visitors a neighborhood receives from residents of *other* neighborhoods. This is an important proxy for the degree of economic and social dynamism of a neighborhood. However, while a high number of unique visitors is a strong indicator of a neighborhood’s dynamism and vitality, it does not *necessarily* reflect its popularity. While previous research has shown that the most desirable neighborhoods often attract the highest number of visitors (Graif et al., 2017), a neighborhood can be highly dynamic and visible without *necessarily* being considered desirable. Caution is therefore necessary to avoid conflating neighborhood activity and visibility with popularity. The primary focus of neighborhood effects research has been to demonstrate that neighborhood characteristics affect a number of socioeconomic outcomes independent of human capital and individual characteristics (Wodtke et al., 2011; Sharkey & Elwert, 2011; Sharkey & Faber, 2014). In this paper, however, we examine the effect of violent crime as a factor in reducing neighborhood activity and vitality. By measuring the short-term effect of gun violence on the number of unique visitors neighborhoods receive, we estimate the effect of these important events on neighborhood activity and vitality. More precisely, we develop and econometrically test the following hypotheses:

#### Hypothesis (1)

Gun violence incidents significantly reduce the number of visits a neighborhood receives.

#### Hypothesis (2)

 The reduction in the number of unique visitors—and hence, the effect size of the gun violence incidents—varies by the number of casualties.

#### Hypothesis (3)

 Neighborhood activity as a social process is heavily racialized. The reduction in the number of unique visitors—and hence, the effect size of the gun violence incidents—varies by neighborhood racial composition.

Building on past scholarship (Osterling 2007; Levy et al., 2020), we hypothesize (1) that well-publicized gun violence incidents reduce the number of visits that the neighborhood where it happened receives. The key mechanism behind such a phenomenon lies in the publicization of an incident and individuals subsequently hearing about the event through the media or personal networks and subsequently tending, consciously or unconsciously, to spend less time in the given neighborhood. For gun violence incidents that receive little attention (i.e., a single gunshot that results in no injuries or deaths and little police attention), we would expect to have little effect on visits. On the other hand, we also expect harmful gun violence incidents—particularly those resulting in injury or death that also receive substantial police and media attention—to reduce the volume of unique visitors. Our analyses are animated by these hypotheses (2) aimed at examining the effect of gun violence incidents with *different* numbers of casualties, which past research suggests is relevant for media coverage (Kaufman et al., 2020). While death and more victims may increase the media attention an incident receives, theoretically reducing visits, other research suggests that the sites of especially tragic crimes may attract more visits (Magano et al., 2022). Finally, we (3) stratify our analyses based on neighborhood racial composition. Our motivation to stratify our analyses stems from the potential heterogeneity in the effect of gun violence incidents on neighborhood visits based on the neighborhood’s racial composition. In line with this, one proposition may be that gun violence incidents may have little to no impact on visits when they occur in majority-Black or majority-Hispanic neighborhoods since these neighborhoods may be more violent or already perceived to be more violent, for that matter. Thus, gun violence incidents may have little effect on the unique number of visits these neighborhoods receive, given that they may not change how potential visitors perceive the neighborhood. It can also be the case that if a Black or Hispanic neighborhood is not perceived as violent or unsafe before a gun violence incident, the gun violence incident may radically stimulate a change in opinion as potential visitors use the incident as confirmation of stereotypes they have about how unsafe Black or Hispanic neighborhoods are.

## Data

We draw on three publicly available data sources. Gun violence incidents in the United States localities for 2019 are obtained by scraping publicly available data from the ‘Gun Violence Archive.’ This data provides information on the location of gun violence incidents, the number of injuries, fatalities resulting from them. It is worth noting that a vast proportion of Gun Violence Archive data is drawn from publicly available sources, 1which suggests that only incidents that have received some degree of publicity are included. 2After scraping, we match gun violence incidents with neighborhood shapefiles drawn from the US Census in order to geocode all incidents to census block groups.^3^

Our mobility (from neighborhood to neighborhood) data is obtained from SafeGraph, a US firm specializing in mobility and traffic data. SafeGraph aggregates anonymized, repeatedly measured location data from a nationally representative sample of 45 million smartphone devices that is provided by Veraset. We specifically rely on Safegraph’s “Social Distance Metrics” dataset, which provides daily information on individuals’ visits to and from census block groups for every day in 2019. A visit is defined as a cluster of proximal location pings with a duration longer than 1 min. Individual devices may not count for multiple visitors to the same neighborhood on the same day. Home location for a device is determined by SafeGraph using machine learning as the common nighttime (6:00 pm to 7:00 am) location of the device.

### Independent Variables

Our independent variables are obtained from the 2015–2019 American Community Survey (ACS). We draw on the following variables for all US census block groups from the ACS including: (1) logged population density; (2) median age; (3) percent of male residents that are aged between 18 and 34; (4) percent of households that are owner-occupied, percentage of households that have been occupied continuously since 1999; (5) percent non-Hispanic Black population; (6) percent Hispanic population; (7) residential disadvantage index; (8) in-degree disadvantage (see Levy et al., 2020) as well as spatial lag.^4^ More information on our matching approach can be found in the Supplementary Information (SI).

We also consider different types of neighborhoods based on their racial composition. We operationalize predominately White neighborhoods as census block groups that are at least two-thirds non-Hispanic White. We operationalize majority-Black and majority-Hispanic neighborhoods as census block groups that are at least 50% non-Hispanic Black and 50% Hispanic, respectively. These operationalizations have precedence in previous research (Albrecht et al., 2005), and align with the categories past research on mobility patterns (Vachuska, 2023) and violence has focused on (Levy et al., 2020). Moreover, these neighborhoods encompass the vast majority of all gun violence incidents, and the remaining neighborhoods are too heterogeneous in composition to constitute a theoretically meaningful category (Flanagin et al., 2021). While the predominately White neighborhoods in the sample have a wide range of levels of residential disadvantage, the majority-Black and majority-Hispanic neighborhoods in this study overwhelmingly have high levels of residential disadvantage. Precisely because of such a wide range of economic (dis)advantage across neighborhoods, we specifically separate predominately-White neighborhoods into two different categories: high- and low-disadvantage neighborhoods. That is to say, we split the two groups based on whether their level of disadvantage is above or below the mean level of disadvantage for all US neighborhoods. Following past scholarship (Wodtke et al., 2016; Sharkey & Elwert, 2011; Levy et al., 2020), we operationalize residential disadvantage by conducting principal factor analysis over seven variables: percent of residents in poverty, unemployment rate, percent of households that are single-headed, percent of households that receive public assistance, percent of adults over age 25 without a high school diploma, percent of adults over age 25 with a bachelor’s degree or higher, percent of workers in managerial or professional occupations (Levy et al., 2020; Wodtke et al., 2016). In auxiliary analysis, we additionally take into account the “high percent-Black” and “high percent-Hispanic” neighborhoods, which we respectively operationalize as neighborhoods that are at least 80% Black and 80% Hispanic.

### Dependent Variable

We use the 5-year estimates from the American Community Survey in combination with SafeGraph’s Social Distance Metrics to predict the number of visits to a neighborhood in the 28 days before and after a shooting occurs. For each census block group in the U.S., SafeGraph reports the daily counts of devices visiting it from each census block group. We calculate the average daily number of visits (SafeGraph devices) to a neighborhood in 2019 using a multiplier to account for inter-neighborhood and inter-day variation in the ratio of residential population to device count, as shown in Eq. (1) below. Notably, this approach accounts for the systematic underrepresentation or overrepresentation of certain geographic groups in the SafeGraph panel, which has been suggested as a potential concern of SafeGraph data (Squire, 2019).

1 $${V}_{jd}=\sum \frac{{v}_{ijd}*{p}_{i}}{{n}_{id}}$$

${V}_{jd}$ denotes the average number of daily visitors to neighborhood $j$ on day $d$; ${v}_{ijd}$ is the number of visitors to neighborhood $j$ on day $d$ who reside in neighborhood *i*; ${p}_{i}$ is the population estimate for neighborhood *i* that is drawn from the 2015–2019 American Community Survey estimates; and ${n}_{id}$ is the number of SafeGraph devices whose home location was in neighborhood $i$ on day $d$. Importantly, we calculate the number of visitors of specific races by weighting the racial composition of the sending neighborhoods.

2 $${M}_{j}=\frac{\sum \sum {v}_{ijd}*{m}_{i}}{\sum \sum {v}_{ijd}}$$

We also calculate the number of visitors of that specific race to a specific neighborhood as ${M}_{j}$, where ${v}_{ijd}$ is the number of visitors to neighborhood $j$ on day $d$ who reside in neighborhood *i.* For a given race, ${m}_{i}$ denotes the proportion of residents in a neighborhood of that given race.

## Methods

### Adjusted Interrupted Time Series Method

We draw on the Adjusted Interrupted Time Series (AITS) method to estimate the effect of gun violence incidents on the number of unique visitors neighborhoods receive. As a similar design to the Difference-in-Difference (D&D) method, the AITS method has been used in previous research to measure the *causal* effect of community interventions on neighborhood real estate values (Galster et al., 1999). It is important to note that the AITS analysis provides a powerful tool to isolate the causal impact of interventions because it allows us to estimate a slope effect in addition to a level effect. AITS is considered a valuable technique for isolating the causal effect of interventions in longitudinal data (Deng & Freeman, 2011). The AITS technique falls under the class of matched treatment–control quasi-experimental methods, requiring us to identify a control group of neighborhoods that did *not* experience the intervention in the relevant period for which each treatment neighborhood did. It must not go unnoticed that our choice to use the AITS method over alternative options such as Regression Discontinuity (RD), Difference-in-Difference (D&D), and Event Study (ES) was not arbitrary. AITS’ flexible assumptions are appropriate, given our research design. In the Supplementary Information (SI), we detail the justifications for the advantages of the AITS technique over the alternatives. It should be noted that a unique advantage of the AITS technique is that the often-violated parallel trends assumption is not necessary, and that the model explicitly adjusts for treatment–control differences in pre-intervention trends.

In our study design, an “intervention” is a single^5^ gun violence incident in a given neighborhood. We consider the pre-intervention period to be the 28 days prior to the shooting and the post-intervention period to be the 28 days after the shooting. We limit the time period to four weeks before and after a gun violence incident to allow sufficient time to estimate linear trends in visitors’ mobility patterns but not so much to unnecessarily increase the likelihood of the trend being contaminated^6^ by other potential incidents. For each unique incident, we identify a control neighborhood, similar to the treatment neighborhood where the incident occurred, but where absolutely no gun violence incidents occurred during the pre- or post-intervention period. We identify the best control neighborhoods based on a set of variables that have been shown to be strongly predictive of a neighborhood’s level of violent crime.^7^ By identifying neighborhoods that did not experience a shooting but were essentially just as likely to, we can minimize selection bias and comfortably assume any difference in the post-intervention difference in the two neighborhood’s intercept and slope is the effect of the intervention.^8^ While this, like most assumptions, is imperfect, we believe it to be quite reasonable and a fairly optimal approach given the available data.

The AITS technique estimates two average effects of the intervention, which is a gun violence incident in our study design. First, the effect of the intervention on the level of the outcome variable: the logged number of unique visitors. A significant effect here implies the intervention causes an immediate change in the number of visitors neighborhoods receive. Second, a potential effect of the intervention is a change in the slope of the outcome variable over time. In this context, a significant effect implies the intervention yields a long-term change in the trend of the number of visitors neighborhoods receive.

### Model Specification

Models derived from the AITS technique entail three relatively straightforward assumptions. First, we assume that both the pre-and post-intervention slopes of visitors over time are linear. Second, we assume the matched-treatment counterfactual holds true for any changes in the level of the outcome variable. More specifically, if not for the gun violence incident, the treatment would experience a change in pre-intervention to post-intervention level that is equivalent to the control neighborhood’s change. The third assumption is similar; we assume the matched-treatment counterfactual holds true for any changes in the level of the outcome variable. We take great caution in ensuring the principal assumption of AITS—the linearity of pre- and post-interventions remains a reasonable proposition. Since mobility patterns vary considerably by day of the week (for example, more minutes are spent at home on weekends), we set our panel variable not just as daily observations for a specific neighborhood but as daily observations for a specific day of the week in a given neighborhood. This strategy allows us to estimate the linear trends, not from one day to the next, but from one weekday in a given week to that weekday of the following week. We expect this type of panel data to follow linear trends much more closely.

We additionally control for single-day effects on the day before, day of, and day after each gun violence incident. Gun violence is more common on certain days of the week, on certain holidays, and even on days that experience certain weather conditions, phenomena of which all may be associated independently with certain mobility patterns and behaviors (Bridges, 2004; Cheatwood, 1995; Lester, 1979). This suggests that we should also take into account the possibility that days when gun violence incidents are more likely to occur may also have distinct mobility patterns and that such distinct mobility patterns may be even more pronounced or dampened in the specific neighborhoods that end up experiencing a gun violent incident. The day of and after a shooting may also feature a large number of additional visitors, potentially from the emergency response and subsequent investigation by law enforcement and even from members of the media. We control for all these potential distinct mobility patterns by including indicators for the days before, during, and after the intervention in our model. The model can be formally represented in Eq. (1) below:

3 $${\text{ln}(V}_{it})={\alpha }_{0}+{\alpha }_{1}{T}_{i} + {\alpha }_{2}{TR}_{it}+ {\alpha }_{3}{{T}_{i}\cdot TR}_{it}+{\partial }_{4}T\cdot {PRE}_{t}+{\gamma }_{1}{DUR}_{t}+{\gamma }_{2}{T}_{i}\cdot {DUR}_{t}+{\mu }_{1}{NEXT}_{t}+{\mu }_{2}{T}_{i}\cdot {NEXT}_{t}{ + \beta }_{1}{POST}_{t}{ + \beta }_{2}{T}_{i}\cdot {POST}_{t} { + \beta }_{3}{TR}_{it}\cdot {POST}_{t}{ + \beta }_{4}{T\cdot TR}_{it}\cdot {POST}_{t}+ \delta {\text{ln}(WM}_{it})+ {\varepsilon }_{it}$$

In the equation above, ${V}_{it}$ is the number of visitors to neighborhood $i$ on day $t, {T}_{i}$ denotes whether or not the observation is in the treatment group (coded 1 if belongs to the ‘treatment group,’ 0 if otherwise); ${TR}_{it}$= 0 on the day of the shooting, − 1 on the day before, + 1 after, and so on; ${PRE}_{t}$ = 1 if observed value occurs on the day immediately before the shooting; zero otherwise; ${DUR}_{t}$ = 1 if observed value occurs *during* the day of the “treatment”; zero otherwise; ${NEXT}_{t}$ = 1 if observed value occurs on the day immediately after the shooting; zero otherwise; ${POST}_{t}$ = 1 if observed value occurs any day (up to 28 days) after the shooting zero otherwise; $ln {WM}_{it}$ = logged mean visitors that day in contiguous neighborhoods (spatial lag); ${\varepsilon }_{it}$ = an error term with the usual assumed statistical properties for panel linear models given our above-outlined panel variable formulation.

For interpretability, we subsequently estimate the absolute and relative effect of a gun violence incident on visitors using the subsequent formula in Eqs. (4) and (5):

4 $$AE=\sum_{k=2}^{28}{e}^{{\alpha }_{1}+{k*\alpha }_{2}+{k*\alpha }_{3}+{\beta }_{1}+{\beta }_{2}+{k*\beta }_{3}+{k*\beta }_{4}+\delta *{WM}_{\mu }}-\sum_{k=2}^{28}{e}^{{\alpha }_{1}+{k*\alpha }_{2}+{k*\alpha }_{3}+{\beta }_{1}+{k*\beta }_{3}+\delta *{WM}_{\mu }}$$

5 $$RE=\frac{\sum_{k=2}^{28}{e}^{{\beta }_{2}+{k*\beta }_{4}}}{27}$$

We graphically depict pre-intervention trends (and counterfactual post-intervention trends) using the follow formula:

6 $${Y}_{k}={e}^{{\alpha }_{1}+{k*\alpha }_{3}}$$

We graphically depict the post-intervention trend using the follow formula:

7 $${Z}_{k}={e}^{{\alpha }_{1}+{k*\alpha }_{3}+{\beta }_{2}+{k*\beta }_{4}}$$

where *k* is the day being estimated.

Table 1 exhibits the summary statistics for our dependent and independent variables across the final analytical panel dataset. The variables map to all the AITS specification terms, as well as the neighborhood characteristics we stratify models on. Visits refer to our outcome variable. Visits (spatial lag) refer to a spatially lagged outcome variable. ‘Treatment’ refers to a binary variable indicating if an observation is a treated neighborhood or not. ‘Trend’ refers to an integer variable indicating the number of days prior to the shooting the observation refers to. ‘During’, ‘after’, and ‘before’ refer to indicator variables indicating whether or not the ‘trend’ variable is equal to 0, + 1, and − 1. ‘Disadvantage’, ‘prop. White’, ‘prop. Black’, and ‘prop. Hispanic’ refer, respectively, to the level of residential disadvantage, proportion of residents who are non-Hispanic White, proportion of residents who are non-Hispanic Black, and proportion of residents who are Hispanic (of any race).

**Table 1:** Statistics

Variables	N	Mean	Std. Dev	Min	Pctl. 25	Pctl. 75	Max
Visits	4,266,176	1081	2217	0.05	259	1081	734,621
Visits (Spatial Lag)	4,266,176	1049	1072	11	444	1273	32,022
Treatment	4,266,176	0.5	0.5	0	0	1	1
Trend	4,266,176	0.01	16	− 28	− 14	14	28
During	4,266,176	0.02	0.14	0	0	0	1
After	4,266,176	0.49	0.5	0	0	1	1
Before	4,266,176	0.49	0.5	0	0	1	1
Disadvantage	4,209,824	0.63	0.99	− 2.6	− 0.037	1.3	4.8
Prop. White	4,261,011	0.39	0.32	0	0.083	0.68	1
Prop. Black	4,261,011	0.36	0.34	0	0.043	0.66	1
Prop. Hispanic	4,261,011	0.18	0.24	0	0.013	0.26	1

## Results

**Fig. 1:**
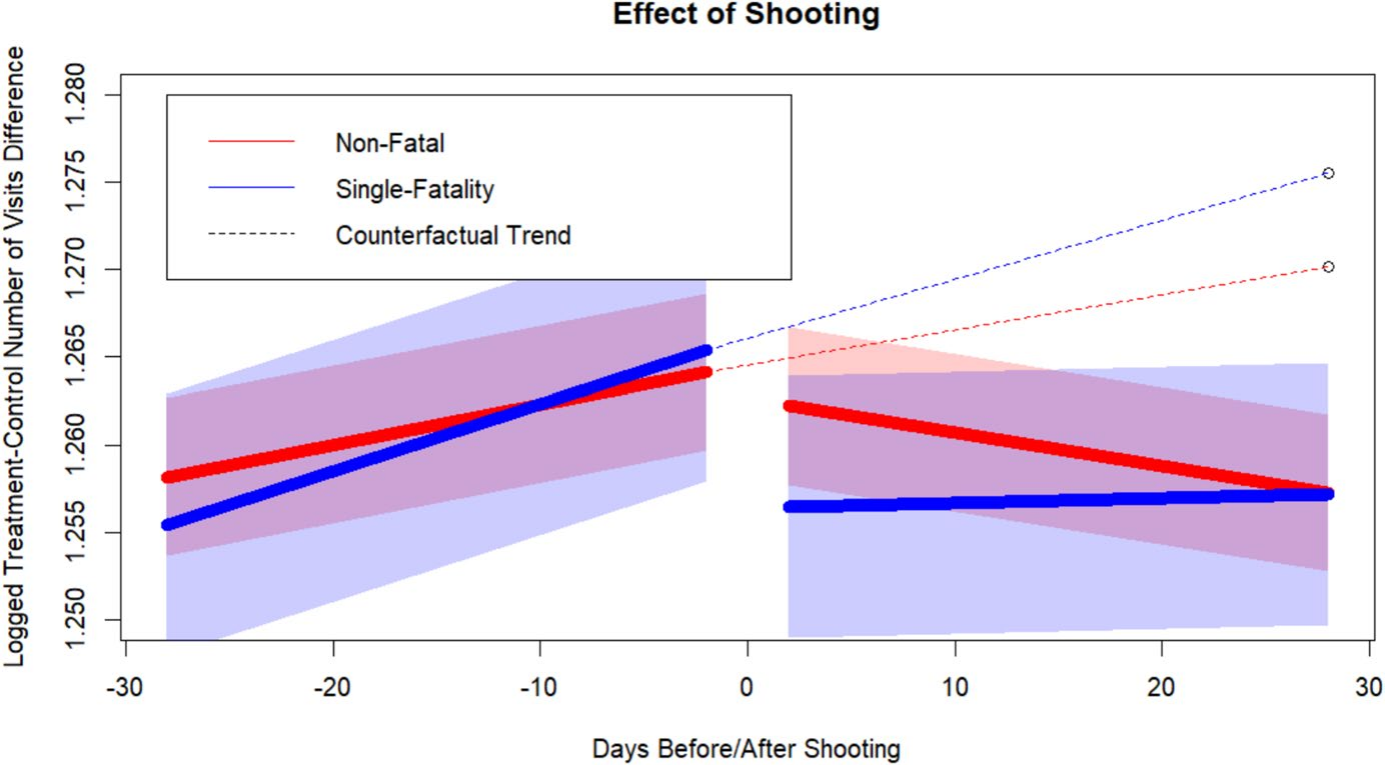
Effect of non-fatal and single fatality shooting

Our main results for gun violence incidents across all neighborhoods are presented in Table 2. The effect of gun violence incidents on the number of unique visitors a neighborhood receives from residents of other neighborhoods is captured in the coefficients for $$T*POST$$ (the level effect) and $$T*TR*POST$$ (the slope effect). For non-fatal shootings, we find a negative, but non-significant level effect. Figure 1 visually displays the average treatment effect, with the solid lines demonstrating the real estimated trends (except for the day before, the day of, and the day after the shooting), and the dashed lines representing the counterfactual trend (i.e., had the shooting *not* occurred). In the time span leading up to non-fatal shootings, we see that the number of unique visitors to treatment neighborhoods is significantly increasing relative to those of control neighborhoods,^9^ but that the shooting has a strong negative effect on that trend. The average treatment effect of the gun violence incident is represented in a net − 0.61% effect on the number of visitors in the 27-day^10^ period after the shooting. The precise interpretation of the average treatment effect of − 0.61% in terms of an approximate number of individuals who would have otherwise visited a given neighborhood that witnessed a violent incident can be computed using the formula represented in Eq. (4). Using our absolute effect approach, we estimate that in our sample, non-fatal and single-fatality gun violence incidents cost US neighborhoods approximately 5.626 million visitors and 3.328 million visitors, respectively, over just the subsequent 27-day period alone. Accordingly, we find strong statistical support for Hypothesis (1): gun violence incidents significantly reduce the number of unique visitors a neighborhood receives.

**Table 2:** All neighborhoods

		No fatalities	One fatality	Two fatalities	Three or more fatalities
Intercept	Intercept	3.164***	3.293***	2.996***	2.628***
T	Treatment (Shooting) Dummy	0.2344***	0.2354***	0.1337***	0.2940***
POST	All Days Post-Treatment Dummy	0.0001	0.0026	− 0.0047	− 0.0137
TR	Day (Trend 1,2,3,…)	0.0003***	0.0002***	0.0009***	0.0005
DUR	Day of Shooting Dummy	0.0008	0.0033	− 0.0152	0.0341
PRE	Day Before Shooting Dummy	− 0.0007	0.0037	− 0.0224*	0.0317
NEXT	Day After Shooting Dummy	0.0022	0.0027	− 0.0122	0.0098
Spatial Lag	Spatial Lag of Visitors	0.5191***	0.4988***	0.5455***	0.5895***
T*POST	Treatment X Post-Shooting	− 0.0015	− 0.0071***	0.0152*	0.0389*
T*TR	Treatment X Day	0.0002***	0.0003**	− 0.0005	− 0.0013
TR*POST	Treatment X Post-Shooting	0.0001*	0.0002	− 0.0007*	0.0011
T*DUR	Treatment X Day of Shooting	0.0119***	0.0223***	0.0625***	0.0686*
T*PRE	Treatment X Day of Shooting	0.0032	− 0.0032	0.0262*	0.0120
T*NEXT	Treatment X Day After Shooting	0.0004	0.0119**	0.0135	0.0211
T*TR*POST	Treatment X Day X Post-Shooting	− 0.0003***	− 0.0003*	0.0006	0.0001
Number of Obs		2,416,686	817,310	81,858	13,446
Number of Unique Incidents		21,926	7401	728	122
Estimated Effect 2–28 days After		0.993824	0.989003	1.023652	1.041767

*p = 0.05, **p = 0.01, ***p = 0.001

It is important to note that our findings exhibit a larger effect size for gun violence incidents with a single fatality. This coefficient has both significant level and significant slope components and constitutes a net − 1.10% effect size. While neighborhoods that experience both non-fatal and single-fatality shootings have relatively similar pre-intervention trends, neighborhoods that experience single-fatality shootings see a much larger drop-off in the number of visitors post-intervention.

**Fig. 2:**
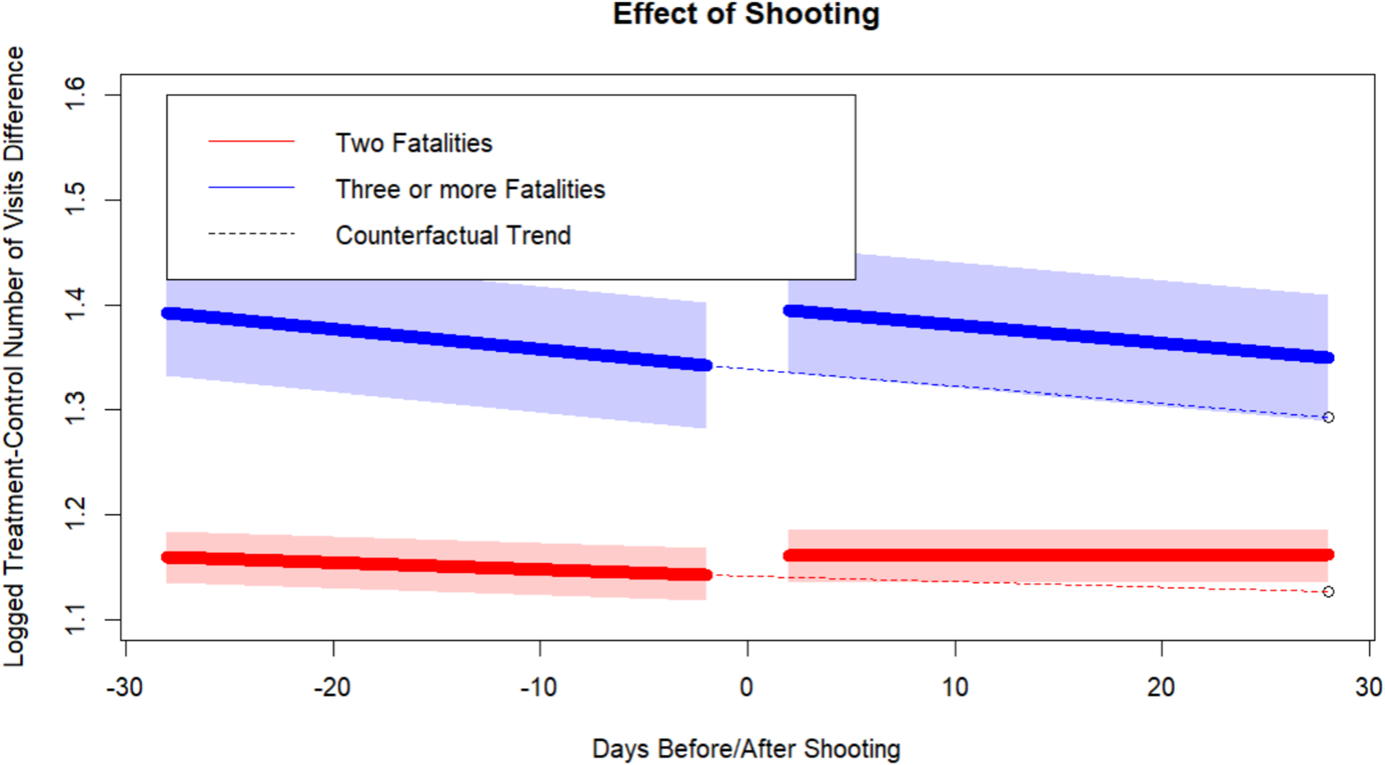
Effect of multiple fatality shooting

The effect of multiple-fatality shootings is strikingly different. Neighborhoods that experience a shooting with 2 or 3 or more fatalities actually see relatively large counterfactual increases in visitors. Our models estimate that a shooting with two fatalities has a positive and significant level effect on the number of subsequent visitors, resulting in a 2.37% increase in the number of visitors in the subsequent 27-day period. Incidents with three or more fatalities have a similar but even larger positive and significant level effect on the number of visitors, resulting in a 4.18% gain in visitors in the subsequent 27-day period. Figure 2 graphically exhibits these treatment effects. Over the subsequent 27-day period after shootings with 2 fatalities and shootings with 3 or more fatalities, treatment neighborhoods received approximately 662,000 and 234,000 more visitors than they would have had the shootings not occurred. As these numbers are positive, in distinction with our earlier result, we find potential support for Hypothesis (2): that the effect of gun violence incidents on mobility varies by the number of casualties.

### Racial and Socioeconomic Heterogeneity

Tables 3 through 6 look at the size of these effects discretely across neighborhoods with different characteristics. These results reveal the only significant effect of non-fatal shootings we actually see is in predominately White neighborhoods. This effect is somewhat larger in predominately White, high-disadvantage neighborhoods (− 2.2%) and operates through highly significant, negative level and slope effects. The effect is much smaller for predominately White low-disadvantage neighborhoods (− 0.8%) and only operates through a slightly significant negative slope effect. Majority-Black neighborhoods experience no impact from non-fatal shootings. Majority-Hispanic neighborhoods experience a small impact (− 0.6%) with a moderately significant negative slope effect. It is crucial to note that the visit patterns to predominately White and high-disadvantage neighborhoods are strongly impacted by non-fatal shootings relative to majority-Black and Hispanic neighborhoods that are often characterized by higher levels of disadvantage. This is a particularly interesting and surprising finding of this study, which suggests that the heterogeneity in the racial composition of neighborhoods has an *independent* effect net of neighborhood socioeconomic status. It should not go unnoticed that less than one-fourth of non-fatal shootings occur in predominately White neighborhoods, but this ratio is much greater in majority-Black and Hispanic neighborhoods, where gun violence is nearly ten times as prevalent.

**Table 3:** Predominately white neighborhoods—low disadvantage

		No fatalities	one fatality	Two fatalities	Three or more fatalities
Intercept	Intercept	2.5253***	2.6022***	2.4419***	1.7617***
T	Treatment (Shooting) Dummy	0.3828***	0.2608***	0.1141**	0.1286
POST	All Days Post-Treatment Dummy	− 0.0036	0.0006	0.0014	0.0973***
TR	Day (Trend 1,2,3,…)	0.0004***	0.0005**	0.0009*	− 0.0038**
DUR	Day of Shooting Dummy	0.0014	− 0.01	− 0.0326	0.1027
PRE	Day Before Shooting Dummy	− 0.0000	0.0049	− 0.0370	0.0906
NEXT	Day After Shooting Dummy	0.0030	− 0.0012	− 0.0398*	0.0472
Spatial Lag	Spatial Lag of Visitors	0.6107***	0.6041***	0.6275***	0.7083***
T*POST	Treatment X Post-Shooting	− 0.0008	− 0.0041	0.0021	− 0.0223
T*TR	Treatment X Day	0.0002	− 0.0002	0.0001	0.0028
TR*POST	Treatment X Post-Shooting	0.0000	− 0.0006*	− 0.0016**	0.0038*
T*DUR	Treatment X Day of Shooting	0.0092	0.0146	0.0584*	− 0.0823
T*PRE	Treatment X Day of Shooting	0.0019	− 0.0087	0.0532	− 0.0713
T*NEXT	Treatment X Day After Shooting	0.0026	0.0065	0.0394	− 0.0777
T*TR*POST	Treatment X Day X Post-Shooting	− 0.0004*	0.0007*	0.0003	− 0.0020
Number of Obs		411,578	110,824	19,084	2622
Number of Unique Incidents		3662	981	168	23
Estimated Effect 2–28 days After		0.992936	1.006271	1.006577	0.948512

*p = 0.05, **p = 0.01, ***p = 0.001

The effect sizes of single-fatality shootings on the number of visitors can also be compared between neighborhoods. Tables 3, 4, 5 and 6 demonstrate the effect size of single fatality shooting is much larger for majority-Black and majority-Hispanic neighborhoods, whereas it is smaller for predominately White neighborhoods. There is even more interesting variation in how much gun violence incidents bear on the frequency of unique visitors among White neighborhoods. Whereas there is evidence of a slight positive slope effect for predominately White low-disadvantage neighborhoods, this effect is null for predominately White high-disadvantage neighborhoods. Majority-Black neighborhoods experience a large negative impact (− 1.9%) on the number of visitors. This result is highly statistically significant and has a negative slope impact. A similar effect size (− 1.8%) with the same direction can be observed for majority-Hispanic neighborhoods as a result of a significant level effect. The importance of these disparities is underscored by the effect that gun violence and single-fatality shootings are vastly more likely to occur in majority Black and Hispanic neighborhoods.

**Table 4:** Predominately white neighborhoods—high disadvantage

		No fatalities	one fatality	Two fatalities	Three or more fatalities
Intercept	Intercept	2.8293***	2.6033***	2.3674***	2.6563***
T	Treatment (Shooting) Dummy	0.2215***	0.2149***	0.1901***	0.4069***
POST	All Days Post-Treatment Dummy	0.0028	0.0075	0.0159	− 0.0343
TR	Day (Trend 1,2,3,…)	0.0002*	− 0.0001	0.0003	− 0.0002
DUR	Day of Shooting Dummy	0.0074	0.0011	0.0081	− 0.0619
PRE	Day Before Shooting Dummy	− 0.0095*	0.0104	− 0.0124	0.0237
NEXT	Day After Shooting Dummy	0.0099*	0.0132	− 0.0111	0.0913
Spatial Lag	Spatial Lag of Visitors	0.5601***	0.5887***	0.6062***	0.5678***
T*POST	Treatment X Post-Shooting	− 0.0089**	− 0.0047	− 0.0133	0.0587
T*TR	Treatment X Day	0.0006***	0.0003	− 0.0009	0.0027
TR*POST	Treatment X Post-Shooting	0.0003*	0.00003	0.0003	0.0030
T*DUR	Treatment X Day of Shooting	− 0.0033	0.0297*	0.0280	0.0951
T*PRE	Treatment X Day of Shooting	0.0122	− 0.0187	0.0063	0.0303
T*NEXT	Treatment X Day After Shooting	− 0.0116	− 0.0091	0.0124	− 0.0911
T*TR*POST	Treatment X Day X Post-Shooting	− 0.0009***	− 0.0001	0.0017	− 0.0055*
Number of Obs		338,596	98,676	13,320	2182
Number of Unique Incidents		3019	881	117	20
Estimated Effect 2–28 days After		0.978206	0.993741	1.01197	0.976113

*p = 0.05, **p = 0.01, ***p = 0.001

**Table 5:** Majority black neighborhoods

		No fatalities	One fatality	Two fatalities	Three or more fatalities
Intercept	Intercept	3.782***	3.735***	3.175***	2.5186***
T	Treatment (Shooting) Dummy	0.0579***	0.0999***	0.0229	0.1950*
POST	All Days Post-Treatment Dummy	0.0019	− 0.0015	0.0028	− 0.0618*
TR	Day (Trend 1,2,3,…)	0.0002*	0.0001	0.0001	0.0045***
DUR	Day of Shooting Dummy	0.0029	0.0043	0.0154	− 0.0100
PRE	Day Before Shooting Dummy	0.0023	0.0028	− 0.0311	− 0.0158
NEXT	Day After Shooting Dummy	0.0020	0.0036	0.0230	0.0162
Spatial Lag	Spatial Lag of Visitors	0.4225***	0.4244***	0.5191***	0.6093***
T*POST	Treatment X Post-Shooting	0.0011	− 0.0047	− 0.0196	0.1173**
T*TR	Treatment X Day	− 0.0000	0.0005**	0.0002	− 0.0047**
TR*POST	Treatment X Post-Shooting	0.0002*	0.0006**	0.0002	− 0.0043*
T*DUR	Treatment X Day of Shooting	0.0182***	0.0343***	0.0364	0.1129
T*PRE	Treatment X Day of Shooting	0.0050	− 0.0023	0.0179	− 0.0060
T*NEXT	Treatment X Day After Shooting	− 0.0048	0.0177*	− 0.0047	− 0.0100
T*TR*POST	Treatment X Day X Post-Shooting	− 0.00008	− 0.0010***	0.0012	0.0042
Number of Obs		629,650	246,436	17,492	2066
Number of Unique Incidents		5855	2279	160	19
Estimated Effect 2–28 days After		0.999963	0.98118	0.99768	1.196254

*p = 0.05, **p = 0.01, ***p = 0.001

**Table 6:** Majority Hispanic neighborhoods

		No fatalities	One fatality	Two fatalities	Three or more fatalities
Intercept	Intercept	3.9461***	4.2209***	4.5724***	3.8289***
T	Treatment (Shooting) Dummy	0.1845***	0.2829***	0.2347***	− 0.0177
POST	All Days Post-Treatment Dummy	− 0.0007	0.0085*	− 0.0370**	− 0.1396***
TR	Day (Trend 1,2,3,…)	0.0005***	0.0002	0.0016**	0.0054***
DUR	Day of Shooting Dummy	− 0.0034	0.0107	− 0.0173	− 0.0541
PRE	Day Before Shooting Dummy	− 0.0054	0.0039	0.0265	0.0386
NEXT	Day After Shooting Dummy	− 0.0041	− 0.0006	0.0141	0.0130
Spatial Lag	Spatial Lag of Visitors	0.4153***	0.3789***	0.3339***	0.4678***
T*POST	Treatment X Post-Shooting	0.0009	− 0.0136**	0.0659***	0.1782***
T*TR	Treatment X Day	0.0002	0.0005*	− 0.0014	− 0.0076***
TR*POST	Treatment X Post-Shooting	0.0000	0.0004	0.0009	0.0013
T*DUR	Treatment X Day of Shooting	0.0090	0.0196*	0.0723*	0.1755
T*PRE	Treatment X Day of Shooting	− 0.0005	0.0071	− 0.0140	0.0311
T*NEXT	Treatment X Day After Shooting	0.0010	0.0156	− 0.0031	0.0788
T*TR*POST	Treatment X Day X Post-Shooting	− 0.0005*	− 0.0003	− 0.0029**	0.0007
Number of Obs		302,640	123,478	10,436	1482
Number of Unique Incidents		2734	1114	93	13
Estimated Effect 2–28 days After		0.993916	0.982081	1.022601	1.207016

*p = 0.05, **p = 0.01, ***p = 0.001

### Race-Specific Visitors

Racial segregation remains deeply entrenched across US localities, and violence is often cited as one of the key drivers of neighborhood and residential segregation (Hipp, 2011). This study examines whether gun violence has a measurable causal effect on mobility-based segregation, where individuals primarily visit neighborhoods that correspond with their racial identity. To better capture this dynamic, we refine our analysis by focusing on neighborhoods with greater racial homogeneity. A higher threshold for defining neighborhood homogeneity allows us to identify more racially uniform areas. By conducting our analysis on these more homogeneous neighborhoods, we can draw stronger inferences about the racialization of mobility patterns and the role of violence in reinforcing segregation.

To operationalize this analytical strategy, we explore the effect of gun violence on race-specific mobility patterns, focusing on neighborhoods with high racial homogeneity. To empirically assess this relationship, we examine the effect of gun violence on race-specific visitor flows, focusing on neighborhoods where Black and Hispanic residents comprise at least 80% of the population. The choice to examine these neighborhoods is not arbitrary; it is based on the fact that they are significantly more homogenous in terms of their demographic composition and thus are more likely to be racialized by outsiders, allowing us to tease out and analyze *race-specific* visit pattern effects that may not necessarily be present in more heterogeneous neighborhoods. We limit this analysis to shootings with one fatality, given that we find the largest overall effect for majority-Black and Hispanic neighborhoods in single fatality incidents.

Table 7 shows estimated effects across visitors from all racial groups: White visitors, Black as well as Hispanic visitors. We see a slightly larger overall effect size in this model compared to the ones operationalized for majority-Black and majority-Hispanic neighborhoods previously, with the 27-day effect estimated at − 2.80% and − 3.82% for majority-Black and majority-Hispanic neighborhoods, respectively. Looking at White visitors alone, we see far greater effect sizes, with models indicating that single-fatality shootings in high percent-Black neighborhoods and high percent-Hispanic neighborhoods exhibit − 3.34% and − 6.26% effect sizes on the number of White visitors to that neighborhood.^11^ Consistent with Hypothesis (3) outlined earlier, we find that, similar to other socioeconomic outcomes that exhibit racialized distributions—where, for instance, Black individuals consistently experience lower rates of income mobility compared to their White counterparts—neighborhood activity and visibility also follow a *racialized* process. Our strongest evidence for the racialized processes at play is represented in the changes in visit patterns in the aftermath of gun violence that tend to be different across racial groups: the visiting patterns of White individuals in Black and Hispanic neighborhoods are significantly influenced compared to the visiting patterns of their Black and Hispanic counterparts. That is to say, when a violent incident occurs, White individuals refrain from visiting those neighborhoods that experienced such incidents at much higher proportion compared to their Black and Hispanic counterparts.

**Table 7:** High percent-black and high percent-Hispanic neighborhoods

	Very Black Neighborhoods			Very Hispanic Neighborhoods		
	All visitors	White visitors	Black visitors	All visitors	White visitors	Hispanic visitors
Intercept	3.8861***	3.1052***	2.6074***	4.3208***	3.2445***	3.2290***
T	0.0472***	− 0.1051***	0.1987***	0.2804***	0.2152***	0.3160***
POST	− 0.005	− 0.0036	− 0.0038	0.0141*	0.0263**	0.0124
TR	0.0002	0.0002	0.0002	− 0.0000	− 0.0004	− 0.0003
DUR	− 0.0002	0.0015	0.0045	0.0202	0.0354*	0.0170
PRE	0.0013	0.0028	0.0065	− 0.0041	0.02360	− 0.0153
NEXT	0.0050	0.0139	− 0.0004	0.0028	0.0076	0.0059
Spatial Lag	0.3925***	0.4009***	0.5184***	0.3561***	0.3573***	0.4629***
T*POST	− 0.0072	− 0.0138	− 0.0059	− 0.0212*	− 0.0465***	− 0.0194*
T*TR	0.0008***	0.0011**	0.0006*	0.0010**	0.0014**	0.0011**
TR*POST	0.0007**	0.0004	0.0005	0.0011**	0.0012*	0.0014***
T*DUR	0.0362**	0.0568***	0.0243	0.0005	0.0108	− 0.0017
T*PRE	− 0.0151	− 0.0251	− 0.0180	0.0069	− 0.0305	0.0202
T*NEXT	0.0211	0.0119	0.0275*	0.0027	0.0213	− 0.008
T*TR*POST	− 0.0014***	− 0.0014**	− 0.0011**	− 0.0012*	− 0.0012	− 0.0013*
Number of Obs	133,560	133,560	133,560	40,588	40,588	40,588
Number of Unique Incidents	1236	1236	1236	366	366	366
Estimated Effect 2–28 Days After	0.972047	0.965997	0.977543	0.961779	0.937363	0.961886

*p = 0.05, **p = 0.01, ***p = 0.001

## Discussion & Conclusion

A substantial body of research demonstrates that neighborhood connectedness—that is, the extent to which neighborhoods are tied to one another by the movement of their residents (Sampson, 2012)—bears on a whole range of outcomes, including crime, health, civic engagement, and leadership networks (Browning et al., 2017; Hipp & Boessen, 2017; Papachristos & Bastomski, 2018; Sampson, 2012). In this vein, three streams of ideas about neighborhood connectedness and integration can be identified. First, the greater the movement of people between neighborhoods, the greater the degree of their social capital (Sampson, 2008, 2012). Second, the greater the movement of residents between neighborhoods, the greater cultural diffusion, such as those manifested in tastes, fashion, values, attitudes, and cultural practices (Brown-Saracino, 2017; Small et al., 2010). Hence, the extent to which people travel between two neighborhoods will shape the extent to which they accept variegated cultural practices (i.e., interracial marriages, cultural festivities, etc.). Third, when neighborhoods are interconnected, different ideas, information, beliefs, and attitudes travel more quickly. Construed in this way, that is, cities as networks, such resources are more likely to be made available to the residents of the neighborhoods.

This paper sought to investigate the direct causal effect of gun violence on neighborhood activity—and, to an extent, popularity. We conceptualized neighborhood activity as the unique number of visitors a neighborhood receives from residents of other neighborhoods. We underscored the fact that neighborhood activity is a crucial dimension of its economic vitality and dynamism. As Wheeler (2021) emphasizes, business establishments pay close attention to the characteristics of neighborhoods—and not necessarily cities—before settling in them (2006). Two main findings stand out. First, we find non-fatal shootings reduce neighborhood visitors in the subsequent 28-day period by − 0.6%, which translates to 5.6 million individuals across our entire sample. That is to say, 5.6 million individuals who would have otherwise visited a neighborhood refrain from doing so given the violent incident. Notably, we found no negative effect of non-fatal gun violence incidents in majority-Black and Hispanic neighborhoods. This is likely the result of either already-existent violent crime saturation or media reporting disparities. Our second main finding is that violent shootings with a single fatality reduce neighborhood visitors by an even greater margin, 1.1% (which translates to 3.3 million individuals) over 28 days. It must not go unnoticed that this is the heterogeneous effect; it is absent in predominately White neighborhoods but is large and highly significant in neighborhoods with large proportions of Black and Hispanic populations (− 2.8% and − 3.8%, respectively).

We find evidence suggesting that the number of visitors to high percent-Black and high percent-Hispanic neighborhoods drops in a greater volume among White non-residents compared to Black and Hispanic non-residents. That said, our results for multiple-fatality shootings depart from the rest that we examined above and are particularly striking. Multiple-fatality shootings actually increase the number of visitors to a neighborhood (+ 2.4 to + 4.2%). It must not go unnoticed that multiple-fatality shootings represent a very small fraction of gun violence in the United States (< 2.5% of all incidents). Future research should investigate this divergence in results between single-fatality compared to multiple-fatality incidents. While we cannot speak to why this inconsistent finding occurs, we think it has to do with how such extreme acts are uniquely perceived by the public and the distinct form of public attention they may generate (Magano et al., 2022). We further emphasize that gun violence incidents with multiple fatalities tend not to be representative of modern urban violence that takes place in disadvantaged neighborhoods regularly and typically involves single targets. Gun violence incidents with multiple victims are far less common and much more likely to constitute indiscriminate rampages committed without another crime being a means to an end. As a result, gun violence incidents with multiple fatalities may be more likely to constitute a spectacle of interest for the public (Kaufman et al., 2020), perhaps attracting attention to the site where it occurred (Magano et al., 2022).

There are several limitations to bear in mind when interpreting the results of this paper, as well as several related possible directions for future research. First, SafeGraph data aggregates visits of all forms—without the ability to distinguish between visits in terms of the discretion individuals have over making them. For example, a homicide in a neighborhood is unlikely to affect mobility in terms of the visitors who work in that neighborhood. Future research that can better disaggregate visit types will shed especially clearer light on the impact of homicide on mobility. In addition, the circumstances of a gun violence incident and the social position of the victim may play a role in how the incident affects mobility patterns. Our dataset lacked relevant measurements of these attributes necessary to examine heterogeneity in this way—with the exception of victim count, which past research suggests is one of the dominant factors associated with media coverage (Kaufman et al., 2020). Other attributes of the gun violence incident, such as the race or sex of the victim, may be especially relevant for understanding heterogeneity in gun violence’s impacts, and future research that can draw on richer data will be especially insightful.

In short, while conceptualizing neighborhood activity as a crucial dimension of the economic and social dynamism that neighborhoods experience, we emphasize that the findings of our study should be located within the broader “neighborhood effect” research focusing on violent crimes. Our research presents several informative findings. We find a negative effect for gun violence incidents—whether with no fatality or just one fatality—on the number of unique visitors a neighborhood receives in the 28 days after the incident. The findings of our research demonstrate that the relationship between violent crime and neighborhood activity is a deeply racialized process. Indeed, one of the central implications of this study for the broader neighborhood research is our finding that demonstrates the vastly *disproportionate* effects of gun violence on neighborhood visibility among predominately-White versus majority-Black and Hispanic neighborhoods. Violent incidents disproportionately punish the latter, which are already marginalized and racialized, both indicative of more social isolation. The novel findings of our research shed further light on the absolute and racialized consequences of the gun violence epidemic. It identified the effect of gun violence on neighborhood visibility. Our estimation results reveal that in 2019, gun violence incidents led to an average loss of nearly 9 million unique visitors for neighborhoods, a tremendous *cost* for neighborhood visibility and activity, and, hence, economic dynamism. Further research should continue to investigate how gun violence affects the fabric of U.S. neighborhoods, and further investments need to be allocated for community social infrastructure that helps mitigate gun violence. Curbing violence is one avenue to increase neighborhood vitality and dynamism, both of which are necessary conditions for local economic development and human flourishing.

## Supplementary Information

Below is the link to the electronic supplementary material.

Supplementary file1 (DOCX 105 KB)

## Data Availability

The data underlying this article cannot be shared due to the terms and conditions of the underlying data, which do not permit re-sharing. The data can be requested from the corresponding author given the explicit permission of SafeGraph.

## Footnotes

1 Gun Violence Archive “utilize automated queries, manual research through over 7500 sources from local and state police, media, data aggregates, government” as well as other credible sources on daily basis. Each gun violence incident reported is verified by multiple validation processes.

2 This feature of the data aligns with the theoretical notion behind this work, because we only expect visiting patterns to be affected if information on gun violence incidents is salient. Thus, we would not expect an unpublicized gun violence incident to affect most people, since potential visitors are unlikely to learn about the incident otherwise. Such a postulation follows logically from a wide-breadth of economic research that has emphasized salience of information in actors’ behavior (Bordalo et al., 2013).

3 Some Gun Violence incidents have a city but no address. Others do not return a best match with a specific address (e.g. just a city center) when we attempt to geocode them. These are excluded from consideration. See Table S1 for more information.

4 In-Degree Disadvantage is a measure created by Levy et al. (2020) that has been found to be more predictive of neighborhood violence than Residential Disadvantage. In-Degree Disadvantage is essentially the average level of Residential Disadvantage in the home neighborhood of visitors to a neighborhood.

5 or multiple incidents, if all are on the same day.

6 Incidents that are within 56 days of another incident in the same neighborhood are excluded from the analysis. This is in line with past applications of the AITS method (Theodos et al., 2024). For a more substantial discussion of this methodological choice and sample comparison, please see the SI section, “Matching Procedure”.

7 Matching based on Mahalanbois distance has been argued to be advantageous over Propensity Score matching. (King & Nielsen, 2019).

8 Please see Supplementary Information (SI) for detailed analysis of the matching procedure.

9 The size of the increase across the entire pre-intervention period only amounts to approximately 1%.

10 We say “27-day” to refer to days 2–28 after the shooting in aggregate. We exclude the day immediately after the shooting because the mobility pattern disruptions in the immediate aftermath of the shooting does not truly represent sustained longer-term shifts in mobility patterns.

11 ${\beta }_{2}$ is significantly larger in both cases.

## References

[CR1] Albrecht, D. E., Albrecht, C. M., & Murguia, E. (2005). Minority concentration, disadvantage, and inequality in the nonmetropolitan United States. *Sociological Quarterly,**46*(3), 503–523.

[CR2] Bordalo, P., Gennaioli, N., & Shleifer, A. (2013). Salience and consumer choice. *Journal of Political Economy,**121*(5), 803–843.

[CR3] Bridges, F. S. (2004). Rates of homicide and suicide on major national holidays. *Psychological Reports,**94*(2), 723–724. 15154207 10.2466/pr0.94.2.723-724

[CR4] Browning, C. R., Calder, C. A., Ford, J. L., Boettner, B., Smith, A. L., & Haynie, D. (2017). Understanding racial differences in exposure to violent areas: Integrating survey, smartphone, and administrative data resources. *The Annals of the American Academy of Political and Social Science,**669*(1), 41–62. 28845047 10.1177/0002716216678167 PMC5567748

[CR5] Brown-Saracino, J. (2017). Explicating divided approaches to gentrification and growing income inequality. *Annual Review of Sociology,**43*, 515–539.

[CR6] Cheatwood, D. (1995). The effects of weather on homicide. *Journal of Quantitative Criminology,**11*(1), 51–70.

[CR7] Chetty, R., Friedman, J. N., Hendren, N., Jones, M. R., & Porter, S. R. (2018). *The opportunity atlas: Mapping the childhood roots of social mobility*. National Bureau of Economic Research.

[CR8] Chiong, K., Kim, S. M., & Tongil Kim T. I. (2025). Mass shootings and their impact on retail. *Marketing Science*.

[CR9] Cobbina, J. E., Miller, J., & Brunson, R. K. (2008). Gender, neighborhood danger, and risk-avoidance strategies among urban African–American youths. *Criminology,**46*(3), 673–709.

[CR10] Cuellar, M., & Jung, H. (2025). Mass Shootings, community mobility, and the relocation of economic activity. arXiv preprint arXiv:2502.19640.

[CR11] Cullen, J. B., & Levitt, S. D. (1999). Crime, urban flight, and the consequences for cities. *Review of Economics and Statistics,**81*(2), 159–169.

[CR12] Deng, L., & Freeman, L. (2011). Planning for evaluation: Using regression discontinuity to evaluate targeted place-based programs. *Journal of Planning Education and Research,**31*(3), 308–318.

[CR13] Farley, R., Steeh, C., Krysan, M., Jackson, T., & Reeves, K. (1994). Stereotypes and segregation: Neighborhoods in the Detroit area. *American Journal of Sociology,**100*(3), 750–780.

[CR14] Ferraro, K. F. (1995). *Fear of crime: Interpreting victimization risk*. SUNY press.

[CR15] Flanagin, A., Frey, T., Christiansen, S. L., & Bauchner, H. (2021). The reporting of race and ethnicity in medical and science journals: Comments invited. *JAMA,**325*(11), 1049–1052. 33616604 10.1001/jama.2021.2104

[CR16] Friedson, M., & Sharkey, P. (2015). Violence and neighborhood disadvantage after the crime decline. *The Annals of the American Academy of Political and Social Science,**660*(1), 341–358.

[CR17] Galster, G. C., Tatian, P., & Smith, R. (1999). The impact of neighbors who use section 8 certificates on property values. *Housing Policy Debate,**10*(4), 879–917.

[CR18] Graif, C., Lungeanu, A., & Yetter, A. M. (2017). Neighborhood isolation in Chicago: Violent crime effects on structural isolation and homophily in inter-neighborhood commuting networks. *Social Networks,**51*, 40–59. 29104357 10.1016/j.socnet.2017.01.007 PMC5663310

[CR19] Grohe, B., Devalve, M., & Quinn, E. (2012). Is perception reality? The comparison of citizens’ levels of fear of crime versus perception of crime problems in communities. *Crime Prevention and Community Safety,**14*(3), 196–211.

[CR20] Harris, D. R. (2001). Why are whites and blacks averse to black neighbors? *Social Science Research,**30*(1), 100–116.

[CR21] Hipp, J. R. (2011). Violent crime, mobility decisions, and neighborhood racial/ethnic transition. *Social Problems,**58*(3), 410–432.

[CR22] Hipp, J. R., & Boessen, A. (2017). The shape of mobility: Measuring the distance decay function of household mobility. *The Professional Geographer,**69*(1), 32–44.

[CR23] Hipp, J. R., Tita, G. E., & Greenbaum, R. T. (2009). Drive-bys and trade-ups: Examining the directionality of the crime and residential instability relationship. *Social Forces,**87*(4), 1778–1812.

[CR24] Jacobs, J. (1961). *The death and life of great American cities*. Random House.

[CR25] Kanan, J. W., & Pruitt, M. V. (2002). Modeling fear of crime and perceived victimization risk: The (in) significance of neighborhood integration. *Sociological Inquiry,**72*(4), 527–548.

[CR26] Kaufman, E. J., Passman, J. E., Jacoby, S. F., Holena, D. N., Seamon, M. J., MacMillan, J., & Beard, J. H. (2020). Making the news: Victim characteristics associated with media reporting on firearm injury. *Preventive Medicine,**141*, 106275. 33027614 10.1016/j.ypmed.2020.106275 PMC7533055

[CR27] Kerr, Z., Evenson, K. R., Moore, K., Block, R., & Roux, A. V. D. (2015). Changes in walking associated with perceived neighborhood safety and police-recorded crime: The multi-ethnic study of atherosclerosis. *Preventive Medicine,**73*, 88–93. 25625690 10.1016/j.ypmed.2015.01.017 PMC4937793

[CR28] King, G., & Nielsen, R. (2019). Why propensity scores should not be used for matching. *Political Analysis,**27*(4), 435–454.

[CR29] Krivo, L. J., Washington, H. M., Peterson, R. D., Browning, C. R., Calder, C. A., & Kwan, M.-P. (2013). Social isolation of disadvantage and advantage: The reproduction of inequality in urban space. *Social Forces,**92*(1), 141–164.

[CR30] Krysan, M. (2002). Community undesirability in black and white: Examining racial residential preferences through community perceptions. *Social Problems,**49*(4), 521–543.

[CR31] Lester, D. (1979). Temporal variation in suicide and homicide. *American Journal of Epidemiology,**109*(5), 517–520. 453175 10.1093/oxfordjournals.aje.a112709

[CR32] Levy, B. L., Phillips, N. E., & Sampson, R. J. (2020). Triple disadvantage: Neighborhood networks of everyday urban mobility and violence in US cities. *American Sociological Review,**85*(6), 925–956.

[CR33] Light, M. T., & Thomas, J. T. (2019). Segregation and violence reconsidered: Do whites benefit from residential segregation? *American Sociological Review,**84*(4), 690–725.

[CR34] Magano, J., Fraiz-Brea, J. A., & Leite, Â. (2022). Dark tourists: Profile, practices, motivations and wellbeing. *International Journal of Environmental Research and Public Health,**19*(19), 12100. 36231400 10.3390/ijerph191912100 PMC9566811

[CR35] Massey, D., & Denton, N. A. (1993). *American Apartheid: Segregation and the making of the underclass*. Harvard University Press.

[CR36] Movahed, M., & Neman, T. (2024). Intergenerational income mobility in the United States: A racial-spatial account. *Social Science Research,**123*, 103064. 39256025 10.1016/j.ssresearch.2024.103064

[CR37] Mrug, S., Madan, A., & Windle, M. (2016). Emotional desensitization to violence contributes to adolescents’ violent behavior. *Journal of Abnormal Child Psychology,**44*(1), 75–86. 25684447 10.1007/s10802-015-9986-x PMC4539292

[CR38] O’Brien, R., Neman, T., Seltzer, N., Evans, L., & Venkataramani, A. (2020). Structural racism, economic opportunity and racial health disparities: Evidence from US counties. *SSM-Population Health,**11*, 100564. 32195315 10.1016/j.ssmph.2020.100564 PMC7076092

[CR39] Osterling, K. L. (2007). Social capital and neighborhood poverty: Toward an ecologically-grounded model of neighborhood effects. *Journal of Human Behavior in the Social Environment,**16*(1–2), 123–147.

[CR40] Papachristos, A. V., & Bastomski, S. (2018). Connected in crime: The enduring effect of neighborhood networks on the spatial patterning of violence. *American Journal of Sociology,**124*(2), 517–568.

[CR41] Peterson, R. D., & Krivo, L. J. (2010). *Divergent social worlds: Neighborhood crime and the racial-spatial divide*. Russell Sage Foundation.

[CR42] Pickett, J. T., Chiricos, T., Golden, K. M., & Gertz, M. (2012). Reconsidering the relationship between perceived neighborhood racial composition and Whites’perceptions of victimization risk: Do racial stereotypes matter? *Criminology,**50*(1), 145–186.

[CR43] Quillian, L., & Midtbøen, A. H. (2021). Comparative perspectives on racial discrimination in hiring: The rise of field experiments. *Annual Review of Sociology,**47*, 391–415.

[CR44] Rader, N. E., May, D. C., & Goodrum, S. (2007). An empirical assessment of the “threat of victimization: ” Considering fear of crime, perceived risk, avoidance and defensive behaviors. *Sociological Spectrum,**27*(5), 475–505. 10.1080/02732170701434591

[CR45] Sampson, R. J. (2008). Moving to inequality: Neighborhood effects and experiments meet social structure. *American Journal of Sociology,**114*(1), 189–231. 10.1086/589843 PMC4211272 25360053

[CR46] Sampson, R. J. (2012). *Great American city*. University of Chicago Press.

[CR47] Sampson, R. J., Morenoff, J. D., & Gannon-Rowley, T. (2002). Assessing “neighborhood effects”: Social processes and new directions in research. *Annual Review of Sociology,**28*(1), 443–478.

[CR48] Sharkey, P., & Elwert, F. (2011). The legacy of disadvantage: Multigenerational neighborhood effects on cognitive ability. *American Journal of Sociology,**116*(6), 1934–1981. 10.1086/660009 PMC3286027 21932471

[CR49] Sharkey, P., & Faber, J. W. (2014). Where, when, why, and for whom do residential contexts matter? Moving away from the dichotomous understanding of neighborhood effects. *Annual Review of Sociology,**40*, 559–579.

[CR50] Small, M. L., Harding, D. J., & Lamont, M. (2010). Reconsidering culture and poverty. *The Annals of the American Academy of Political and Social Science,**629*(1), 6–27.

[CR51] Squire, R. F. (2019). Quantifying sampling bias in SafeGraph patterns. SafeGraph. Retrieved from https://colab.research.google.com/drive/1u15afRytJMsizySFqA2EPlXSh3KTmNTQ#sandboxMode=true&scrollTo=xsNNli6GTN6s&printMode=true.

[CR52] Theodos, B., Galster, G., & Hermans, A. (2024). Neighborhood home price impacts of community development block grant spending. *Cityscape,**26*(3), 25–52.

[CR53] Tokman, A. (2016). *Crime and the geography of the city: Measuring the effect of crime on urban residential*.

[CR54] Vachuska, K. (2023). Racial segregation in everyday mobility patterns: Disentangling the effect of travel time. *Socius,**9*, 23780231231169260.

[CR55] Wang, Q., Phillips, N. E., Small, M. L., & Sampson, R. J. (2018). Urban mobility and neighborhood isolation in America’s 50 largest cities. *Proceedings of the National Academy of Sciences,**115*(30), 7735–7740. 10.1073/pnas.1802537115 PMC6065036 29987019

[CR56] Wheeler, C. H. (2021). *Businesses choose neighborhoods, not just cities: St. Louis Fed*. Saint Louis Fed Eagle. https://www.stlouisfed.org/publications/bridges/winter-20052006/businesses-dont-just-choose-a-city-they-choose-a-specific-neighborhood

[CR57] Wilsonm, J. Q. & Kelling, G. L. (1982). Broken windows: The police and neighborhood safety. *Atlantic Monthly,**249*(3), 29–38.

[CR58] Wilson, W. J. (1987). *The truly disadvantaged: The inner city. The underclass, and public policy* (8th edn.). University of Chicago Press.

[CR59] Wodtke, G. T., Elwert, F., & Harding, D. J. (2016). Neighborhood effect heterogeneity by family income and developmental period. *American Journal of Sociology,**121*(4), 1168–1222. 10.1086/684137 PMC4820764 27017709

[CR60] Wodtke, G. T., Harding, D. J., & Elwert, F. (2011). Neighborhood effects in temporal perspective: The impact of long-term exposure to concentrated disadvantage on high school graduation. *American Sociological Review, 76*(5), 713–736. 10.1177/0003122411420816 PMC3413291 22879678

