# Supplementary material for: The Causal Effect of Gun Violence on Everyday Mobility Patterns Across US Neighborhoods
Source: Spat Demogr. 2025 Jul 16;13(1):7. doi: 10.1007/s40980-025-00139-1 (PMC12267329; doi:10.1007/s40980-025-00139-1)
Supplement: Supplementary file 1 — Supplementary file1 (DOCX 105 KB) [file 40980_2025_139_MOESM1_ESM.docx]

**Supplementary Information for**

**The Impact of Gun Violence on Everyday Mobility Patterns**

This file includes:

Supplementary Text

- Descriptive Statistics
- AITS Justification
- Matching Procedure
- Robustness Checks

Figure S1

Table S1

Table S2

Table S3

Table S4

Table S5

Table S6

Table S7

**Supplementary Text**

**Descriptive Statistics**

Table S1 displays statistics on all Gun Violence Archive incidents in 2019 as scraped from the Gun Violence Archive. The data shows that non-fatal incidents constitute the vast majority of shootings in the United States. Our data shows that gun violence is highly concentrated in Black-majority census block groups. Indeed, almost a third of all the United States gun violence occurs there, even though the Black-majority census block groups make up less than 10% of all census block groups. Clearly, as demonstrated below, White neighborhoods experience disproportionately lower rates of gun violence.

Table S2 presents a simple OLS linear regression predicting a neighborhood’s number of visitors based on it’s Percent NH-Black, Percent Hispanic, Percent Other^[[1]](#footnote-1)^, Population, and County the neighborhood is in. This model indicates that Percent NH-Black and Percent Hispanic both have surprisingly strong negative effects on the number of visitors a neighborhood receives. Put in plain terms, a mere 1% increase in the proportion of either Black or Hispanic residents in a neighborhood is approximately correlated with 1 fewer person visiting that neighborhood in a given day, net of county-fixed effects. This finding suggests neighborhoods with greater proportions of Black and Hispanic residents are disproportionately less popular destinations to begin with.

**AITS Justification**

RD (Regression Discontinuity) and ES (Event Study) are implausible methods for this work, given that differences in the pre-intervention and post-intervention level of our outcome may be the result of broader differences in mobility patterns. (For example, visit totals tend to peak on Friday and drop on the weekend, fall or jump around certain holidays, and decline in Winter.) These unstable patterns necessitate a matched-control design in estimating the causal impact of any intervention on this type of outcome.

D&D (Difference-in-difference) is a reasonable methodological option since it allows us to adjust for post-intervention changes in the level of visitors in a control neighborhood. However, a central assumption of D&D, the parallel trends assumption, is clearly violated across our data. A central flexibility of the AITS model is controlling for the pre-intervention slope differences so as to not require such an assumption. Additionally, D&D strictly assumes the causal impact of an intervention is limited to differences in the post-intervention level, not the slope. Past research strongly suggests that the causal impact of violent crime on neighborhood mobility patterns likely involves gradual rather than just abrupt changes. (Graif et al. 2017) For these reasons, we prefer to employ the AITS method over the D&D method. It should, however, be noted that conclusions akin to those obtained from D&D can still be ascertained from our AITS result. A common approach to handling parallel trends assumption violations is to further assume that pre-existing differences in slope would persist if not for the intervention (Rambachan and Roth 2019). This is essentially an assumption AITS already makes. Thus, the main difference here between AITS and D&D (if one chooses to handle Parallel Trends Assumption violations in this way), is that only changes in level, not changes in slope, could be interpreted as causal effects. Subsequently, this approach would change our results somewhat but would not be enough to fully eliminate significant causal effects across many of our models.

**Matching Procedure**

We began our matching procedure by first dropping any observations (incidents of gun violence) that are contaminated by the presence of the neighborhood having experienced any other gun violence incident in the 55 days before or the 55 days after the target incident.^[[2]](#footnote-2)^ As a result of gun violence incidents tending to be spatially and temporally autocorrelated, this rule reduces our sample of incidents considerably. This is, however, a necessary step in order to accurately assess the effects of a given incident.^[[3]](#footnote-3)^ For each neighborhood that experiences a gun violence incident, we identify a matching control neighborhood. Candidate matches must be within the same commuting zone, but must be at least 10 kilometers from the treatment neighborhood. We set this condition in order to identify our control neighborhoods, which are located in the general region of the treatment neighborhoods, but are far enough away to be unlikely to be affected by the shooting the treatment neighborhood experiences. If a treatment neighborhood has less than 40 neighborhoods that are potential matches based on these two requirements, we discontinue the matching procedure and drop that observation. If a neighborhood has at least 40 potential matches at this point, we calculate potential matches.

Consistent with previous research’s recommendations (King and Nelson 2019), we find matches based on Mahalanbois distance rather than propensity score matching. We calculate Mahalanbois distance to the target neighborhood, based on ten variables that are highly predictive of neighborhood violent crime. Starting with the best match, we see if potential matches have already been used for an overlapping time period, or themselves experienced a shooting of any type within the 55 days before or after the shooting. We choose the highest-ranked match as often as we can.^[[4]](#footnote-4)^ If we cannot find a usable match within the top decile of potential matches, we employ a caliper and drop the treatment neighborhood from our study set.^[[5]](#footnote-5)^ Statistics on the ultimate sample are found in Table Four. The sample mostly resembles the original data with the exception that Predominately White neighborhoods are slightly overrepresented at the expense of Majority Black, Hispanic, and High Disadvantage neighborhoods being slightly underrepresented. We do not believe these sample differences are large enough to substantially affect our results, but in case they do, we also separately examine all our findings across these different neighborhood types.^[[6]](#footnote-6)^

**Robustness Checks**

As a transparent series of robustness checks, we run these analyses in three alternative ways. The first alternative approach we apply is running the same models, but only on pairs of treatment and controls where the selected control was the best match for the treatment, excluding all pairs where the best match was not available and the treatment was forced to be matched with a less desirable control. This approach validates that low match quality is not a cause for concern.

The second alternative approach employed involves setting the trend variable as the number of weeks, not days, before or after the shooting, and dropping observations from the day when the shooting occurred. All models utilized as part of this approach drop the $PRE, DUR,$ and $NEXT$ variables and their interaction terms. We still continue to set the panel variable as a specific day of the week in a specific neighborhood. This robustness check demonstrates that our models are robust to a lack of controls for specific-day effects and addresses concerns about different sets of days of the week having different sets of time variables. The model(s) we subsequently estimate can be symbolically written as:

$${ln(V}_{it})=\alpha_{0}+\alpha_{1}T_{i} + \alpha_{2}{TR}_{it}+ \alpha_{3}{T_{i}\cdot TR}_{it}{+ \beta}_{1}{POST}_{t}{+ \beta}_{2}T_{i}\cdot{POST}_{t} {+ \beta}_{3}{TR}_{it}\cdot{POST}_{t}{+ \beta}_{4}{T\cdot TR}_{it}\cdot{POST}_{t}+ \delta{ln(WM}_{it})+ \varepsilon_{it}$$

where

$V_{it}$ is the number of visitors to neighborhood *i* on day t

$T_{i}$ = 1 if neighborhood is in “treatment group” (where shooting occurred); zero otherwise

${TR}_{it}$ = -1 if on any of the 7 days before the shooting, +1 on any of the 7 day after, etc.

${POST}_{t}$ = 1 if observed value occurs any day (up to 28 days) after the shooting zero otherwise

$ln {WM}_{it}$ = logged mean visits that day in contiguous neighborhoods (spatial lag)

$\varepsilon_{it}$ = an error term with the usual assumed statistical properties for panel linear models given our above-outlined panel variable designation.

Lastly, the third robustness check we employ is to estimate the original models with an alternative matching approach. This alternative approach is otherwise identical to the original but involves matching on a reduced set of core variables: Residential Disadvantage, Residential Disadvantage Spatial Lag, In-Degree Disadvantage, %NH-Black, %Hispanic. Consistent with the first approach, matches are still limited to the same Commuting Zone, but must be at least 10 KM away and still must be in the top decile of matches.

Tables S5, S6, and S7 display robustness check model results for our main two results (non-fatal and single-fatality shootings across all neighborhoods. All three sets of results are very similar to our original models and demonstrate the robustness of our main findings.

**Figure S1.**


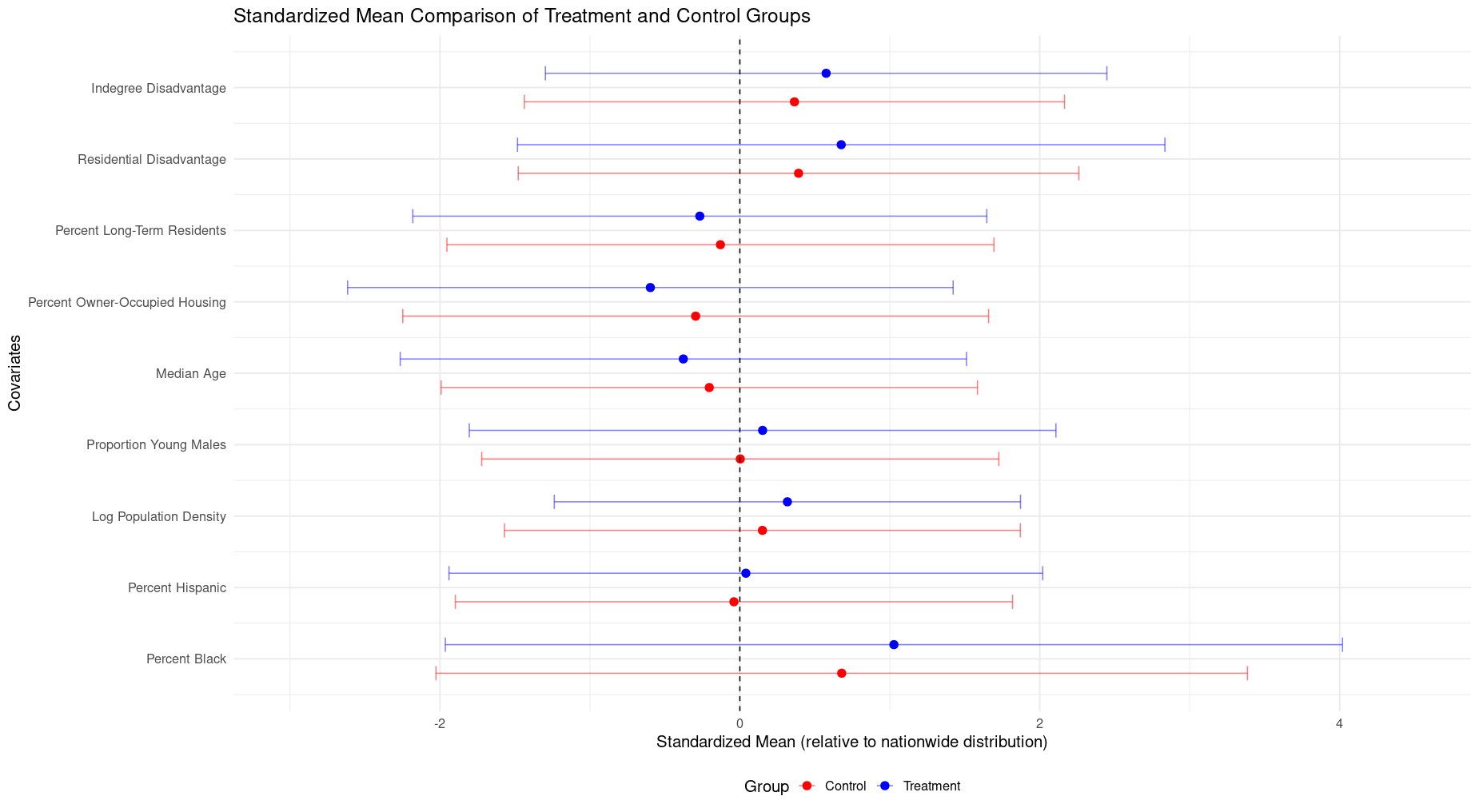


**Table S1 – Gun Violence Incidents by Neighborhood 2019**

|  | Total Shootings | No Fatalities | One Fatality | Two Fatalities | 3-5 Fatalities | Percentage of All Shootings | Percentage of All Neighborhoods |
| --- | --- | --- | --- | --- | --- | --- | --- |
| All | 53476 | 39689 | 12485 | 1118 | 184 |  |  |
| Predominately White | 12352 | 9408 | 2505 | 378 | 61 | 23.10% | 54.90% |
| Majority Black | 17335 | 12535 | 4495 | 272 | 33 | 32.40% | 8.70% |
| Majority Hispanic | 6107 | 4284 | 1672 | 130 | 21 | 11.40% | 10.10% |
| Other | 14003 | 10513 | 3152 | 277 | 61 | 26.20% | 25.80% |
| Not able to geocode | 1829 | 1591 | 223 | 13 | 2 | 3.40% |  |

Table S2 – Neighborhood and Visits

|  | Average Daily Visitors | Visitor % White | Visitor % Black | Visitor % Hispanic |
| --- | --- | --- | --- | --- |
| Predominately White | 1874.568 | 0.758759 | 0.076338 | 0.096831 |
| Majority Black | 1494.696 | 0.408683 | 0.410027 | 0.121533 |
| Majority Hispanic | 2062.092 | 0.296589 | 0.091392 | 0.526073 |
| Other | 2618.681 | 0.532251 | 0.128718 | 0.205538 |

Table S3 – Effect of Race on Visits

|  | Estimate | Std. Error | t value | Pr(>\|t\|) |
| --- | --- | --- | --- | --- |
| (Intercept) | 634.9575 | 45.80922 | 13.86091 | 1.14E-43 |
| Percent Black | -124.522 | 31.43998 | -3.96063 | 7.48E-05 |
| Percent Hispanic | -108.438 | 33.42368 | -3.24435 | 0.001177 |
| Percent Other | 1641.412 | 59.41445 | 27.62649 | 1.05E-167 |
| Population | 0.879439 | 0.006662 | 132.0006 | 0 |
|  |  |  |  |  |
| Number of Obs. | 216684 |  |  |  |

*County level effects not shown

Table S4 – Sample Statistics

|  | All | Predominately White | Majority Black | Majority Hispanic | Top Quartile RD | Top Decile RD |
| --- | --- | --- | --- | --- | --- | --- |
| All – Number | 51647 | 12352 | 17335 | 6107 | 26974 | 14945 |
| All – Percent |  | 23.90% | 33.50% | 11.80% | 52.20% | 28.90% |
| Final Sample- Number | 30177 | 8932 | 8313 | 3954 | 14529 | 7581 |
| Final Sample- Percent |  | 29.60% | 27.50% | 13.10% | 48.10% | 25.10% |

Table S5 - Robustness Check (1)

|  | No Fatalities |  | One Fatality |  |
| --- | --- | --- | --- | --- |
|  | Main Approach | Robustness Check #1 | Main Approach | Robustness Check #1 |
| Intercept | 3.16466*** | 3.06068*** | 3.29355*** | 3.24162*** |
| T | 0.23441*** | 0.27146*** | 0.23541*** | 0.25290*** |
| POST | 0.00007 | 0.00047 | 0.00259 | 0.00359* |
| TR | 0.00029*** | 0.00025*** | 0.00022*** | 0.00017* |
| DUR | 0.00083 | 0.00107 | 0.00331 | 0.00116 |
| PRE | -0.00071 | -0.00155 | 0.0037 | 0.00218 |
| NEXT | 0.00223 | 0.00246 | 0.00265 | 0.00409 |
| Spatial Lag | 0.51906*** | 0.53405*** | 0.49878*** | 0.50807*** |
| T*POST | -0.00153 | -0.00204 | -0.00712*** | -0.00863*** |
| T*TR | 0.00017*** | 0.00023*** | 0.00028** | 0.00036*** |
| TR*POST | 0.00011* | 0.00013* | 0.00016 | 0.00011 |
| T*DUR | 0.01190*** | 0.01144*** | 0.02234*** | 0.02168*** |
| T*PRE | 0.0032 | 0.00371 | -0.00318 | -0.00124 |
| T*NEXT | 0.00043 | 0.0008 | 0.01185** | 0.00573 |
| T*TR*POST | -0.00031*** | -0.00038*** | -0.00026* | -0.00012 |
|  |  |  |  |  |
| Number of Obs. | 2416686 | 1875404 | 817310 | 646886 |
| Number of Unique Incidents | 21926 | 16796 | 7401 | 5774 |
|  |  |  |  |  |
| Estimated Effect 2-28 days After | 0.993823809 | 0.99225031 | 0.989002994 | 0.989556391 |

Table S6 – Robustness Check (2)

|  | No Fatalities | | One Fatality | |
| --- | --- | --- | --- | --- |
|  | Main Approach | Robustness Check #2 | Main Approach | Robustness Check #2 |
| Intercept | 3.16466*** | 3.15721*** | 3.29355*** | 3.28620*** |
| T | 0.23441*** | 0.23509*** | 0.23541*** | 0.23579*** |
| POST | 0.00007 | -0.0017 | 0.00259 | 0.00075 |
| TR | 0.00029*** | 0.00200*** | 0.00022*** | 0.00173*** |
| DUR | 0.00083 |  | 0.00331 |  |
| PRE | -0.00071 |  | 0.0037 |  |
| NEXT | 0.00223 |  | 0.00265 |  |
| Spatial Lag | 0.51906*** | 0.52017*** | 0.49878*** | 0.49994*** |
| T*POST | -0.00153 | -0.00177 | -0.00712*** | -0.00615** |
| T*TR | 0.00017*** | 0.00125*** | 0.00028** | 0.00187** |
| TR*POST | 0.00011* | 0.00067 | 0.00016 | 0.00071 |
| T*DUR | 0.01190*** | | 0.02234*** | |
| T*PRE | 0.0032 |  | -0.00318 |  |
| T*NEXT | 0.00043 |  | 0.01185** | |
| T*TR*POST | -0.00031*** | -0.00228*** | -0.00026* | -0.00214* |
|  |  |  |  |  |
| Number of Obs. | 2416686 | 2416686 | 817310 | 817310 |
| Number of Unique Incidents | 21926 | 21926 | 7401 | 7401 |
|  |  |  |  |  |
| Estimated Effect 2-28 days After | 0.993824 | 0.992567 | 0.989003 | 0.988561 |

Table S7 – Robustness Check (3)

|  | No Fatalities |  | One Fatality |  |
| --- | --- | --- | --- | --- |
|  | Main Approach | Robustness Check #3 | Main Approach | Robustness Check #3 |
| Intercept | 3.16466*** | 3.14755*** | 3.29355*** | 3.30059*** |
| T | 0.23441*** | 0.23962*** | 0.23541*** | 0.23824*** |
| POST | 0.00007 | 0.0013 | 0.00259 | 0.00290* |
| TR | 0.00029*** | 0.00025*** | 0.00022*** | 0.00022*** |
| DUR | 0.00083 | 0.00116 | 0.00331 | 0.00143 |
| PRE | -0.00071 | -0.00106 | 0.0037 | 0.00161 |
| NEXT | 0.00223 | 0.00144 | 0.00265 | 0.0025 |
| Spatial Lag | 0.51906*** | 0.52076*** | 0.49878*** | 0.49763*** |
| T*POST | -0.00153 | -0.00281* | -0.00712*** | -0.00769*** |
| T*TR | 0.00017*** | 0.00020*** | 0.00028** | 0.00027** |
| TR*POST | 0.00011* | 0.00009 | 0.00016 | 0.00012 |
| T*DUR | 0.01190*** | 0.01147*** | 0.02234*** | 0.02439*** |
| T*PRE | 0.0032 | 0.0029 | -0.00318 | -0.00114 |
| T*NEXT | 0.00043 | 0.00106 | 0.01185** | 0.01175** |
| T*TR*POST | -0.00031*** | -0.00029*** | -0.00026* | -0.00022 |
|  |  |  |  |  |
| Number of Obs. | 2416686 | 2417796 | 817310 | 819922 |
| Number of Unique Incidents | 21926 | 21953 | 7401 | 7425 |
|  |  |  |  |  |
| Estimated Effect 2-28 days After | 0.993823809 | 0.992923504 | 0.989002994 | 0.989120621 |

1. Other is operationalized as anyone who is not NH-White, NH-Black, or Hispanic of any race [↑](#footnote-ref-1)
2. 55 days is the minimum number of days needed so as to have at least 4 observations of one day of the week on either side for both incidents. [↑](#footnote-ref-2)
3. 11,411 of the 51,747 geocoded Gun Violence incidents violate this rule. Statistics on the final sample of Gun Violence incidents used can be found in Table Four [↑](#footnote-ref-3)
4. We are able to use the best match 69.7% of the time and a top three match 89.7% of the time. [↑](#footnote-ref-4)
5. We randomize the order of treatment neighborhoods before iterating through and attempting to find matches. [↑](#footnote-ref-5)
6. We additionally trim the data so that treatment and controls have observations for the exact same set of days. So, if a treatment is missing data for a particular day, but a control is not, we delete the corresponding day’s data for the control. We perform the same process where a control is missing data but a treatment is not. We additionally delete all treatment and control day-of-week data sequences where there is not four datapoints before the intervention and four datapoints after. [↑](#footnote-ref-6)
